# Increased Risk of Second Primary Malignancy and Mortality at ten Years After Stem Cell Transplant for Multiple Myeloma: An Analysis of 14,532 Patients

**DOI:** 10.7759/cureus.16372

**Published:** 2021-07-13

**Authors:** Brittany Miles, James D Mackey

**Affiliations:** 1 Medical Education, University of Texas Medical Branch, Galveston, USA; 2 Radiology, University of Texas Medical Branch, Galveston, USA; 3 Medical Oncology, Baylor University Medical Center, Dallas, USA

**Keywords:** multiple myeloma, stem cell transplant, second primary malignancies, immunomodulatory imide drugs, trinetx

## Abstract

Background

The landscape for patients with multiple myeloma has improved dramatically over the last 15 years. Immunomodulatory imide drugs (IMiDs) have shown great efficacy in both the setting of initial therapy and as maintenance after autologous stem cell transplant (ASCT). Concern has arisen, however, regarding the risk of second primary malignancies (SPMs) that appear to be associated with the use of IMiD agents. SPMs are a known sequela of multiple myeloma treatment, particularly as a consequence of maintenance lenalidomide status-post stem cell transplant (SCT). The benefit of SCT has become less clear with the utilization of newer, more effective initial therapies.

Objectives

To determine the effect of SCT on SPM risk and overall survival in multiple myeloma patients at 5 and 10 years after treatment initiation.

Methods

We used TriNetX, a global federated health research network providing access to electronic medical records (diagnoses, procedures, medications, laboratory values, genomic information) from approximately 58 million patients in 49 large healthcare organizations. We created two patient cohorts who had all received treatment with thalidomide, lenalidomide, or pomalidomide. One cohort had received SCT while the other had not. Both cohorts were then analyzed for the development of all non-myeloma malignancies which occurred at least one year after initiation of treatment.

Results

At 5 years, SPMs were 5.8% more likely in patients who received stem cell transplant (22.4% vs 16.6%, RR 0.741, p value <0.0001) but 5-year survival favored transplanted patients by 2.38% (64.85% vs 62.474%, p value 0.0044). 10-year survival favored patients who did not receive transplant by 1.44% (42.279% vs 40.838%, p value 0.0279). The Kaplan-Meier curves cross at year 6.

Conclusions

It has previously been shown that the use of alkylating agents in myeloma patients significantly increases the risk of SPM, but that difference had curiously not been shown to have a negative impact on survival. Our analysis shows that this negative survival impact does exist but requires six or more years of follow up to become evident. Recent analyses from studies using older regimens show no overall survival benefit from the use of stem cell transplant. As non-transplant regimens become more effective at producing minimal residual disease (MRD) negativity, it seems that transplantation for myeloma patients will soon be regarded as unnecessary or even detrimental.

## Introduction

The landscape for patients with multiple myeloma has changed dramatically over the last 15 years. Immunomodulatory imide drugs (IMiDs) have shown great efficacy, as both initial therapy and as maintenance after autologous stem cell transplant (ASCT). Concern has arisen, however, regarding the risk of second primary malignancies (SPMs) that appear to be associated with the use of IMiD agents. The first report of second malignancies in multiple myeloma patients was published in 1977, at which time it was suspected that multiple myeloma itself was a risk factor for the development of other cancers [1]. Mailankody et al. reported in 2010 that there was an 8-fold increase in the incidence of acute myeloid leukemia (AML) and myelodysplastic syndrome (MDS) in patients with monoclonal gammopathy of undetermined significance (MGUS), the precursor condition to multiple myeloma [2]. This increased risk of unrelated malignancies in these untreated patients led to the current belief that development of SPMs is a multifactorial process that includes host genetics, environment (exposures), behavior (smoking, alcohol, obesity, diet), myeloma treatment specifics, and disease-related factors [3]. There is a general consensus that the use of IMiD agents after melphalan (or other alkylating agents) exacerbates the risk of second primary malignancies by a multiple of 2- to 3-fold, although a few studies have failed to show this association [4-15]. Numerous studies have shown that despite the increased risk of SPMs, IMiD treatment for multiple myeloma provides conclusive survival benefit that justifies its continued long-term use [16-18]. However, with increasingly efficacious first-line therapies coming available for treatment of multiple myeloma, we believe it is worthwhile to revisit whether treatment with stem cell transplants provide a net benefit, or whether the increased SPM risk from the alkylating agent exposure that they require will result in more harm than good over time.

## Materials and methods

We used TriNetX, a global federated health research network providing access to electronic medical records (diagnoses, procedures, medications, laboratory values, genomic information) from approximately 58 million patients in 49 large healthcare organizations. The TriNetX platform only uses aggregated counts and statistical summaries of de-identified information. No protected health information (PHI) or personal data is made available to the users of the platform. We identified two cohorts of adult multiple myeloma patients who had all received treatment with an IMiD agent: thalidomide, lenalidomide, or pomalidomide. One of the cohorts must have received SCT as part of their treatment, while the other was prohibited from having received it. After balancing the cohorts for age, race, gender, and ethnicity, each contained 7,259 patients. The cohorts were then analyzed for the development of all non-myeloma malignancies which occurred at least one year after treatment initiation. The difference in malignancy rates between the two cohorts is believed to represent the difference in development of second primary malignancies attributable to alkylating agent exposure as a consequence of stem cell transplantation.

## Results

At five years of follow up, transplanted patients had an SPM rate of 20.534% vs 15.579% for patients who did not receive transplant (P value < 0.0001, RR 0.759, CI 0.707,0.814). Five year survival favored the transplanted group (64.85% vs. 62.474%, P value 0.0044). However, at ten years of follow up, SPMs were 5.8% more likely in patients who received stem cell transplant (22.4% vs 16.6%, RR 0.741 (CI 0.693,0.793), P value <0.0001) but overall survival switched to favor patients who did not receive transplant by 1.44% (42.279% vs 40.838%, p value 0.0279) (See Table 1).

**Table 1:** Five- and ten-year SPM rates and survival

	5-Year SPM	10-Year SPM	5-Year Survival	10-Year Survival
Non-Transplanted	15.579%	16.6%	62.474%	42.279%
Transplanted	20.534%	22.4%	64.85%	40.838%

**Figure 1:**
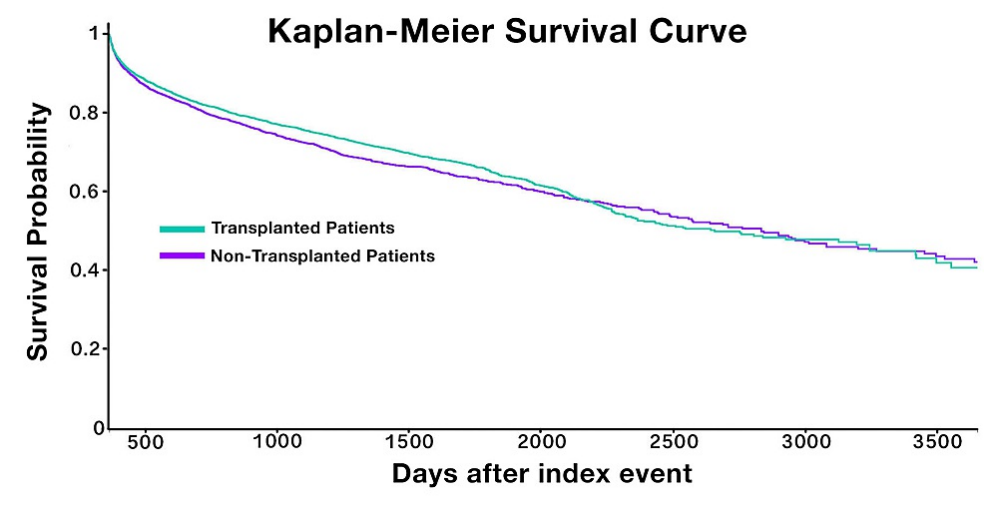
Kaplan-Meier survival curve for patients with and without stem cell transplant

The Kaplan-Meier curves cross during year 6 due to an interesting change in shape of the survival curve of the transplanted group (See Figure 1).

## Discussion

**Figure 2:**
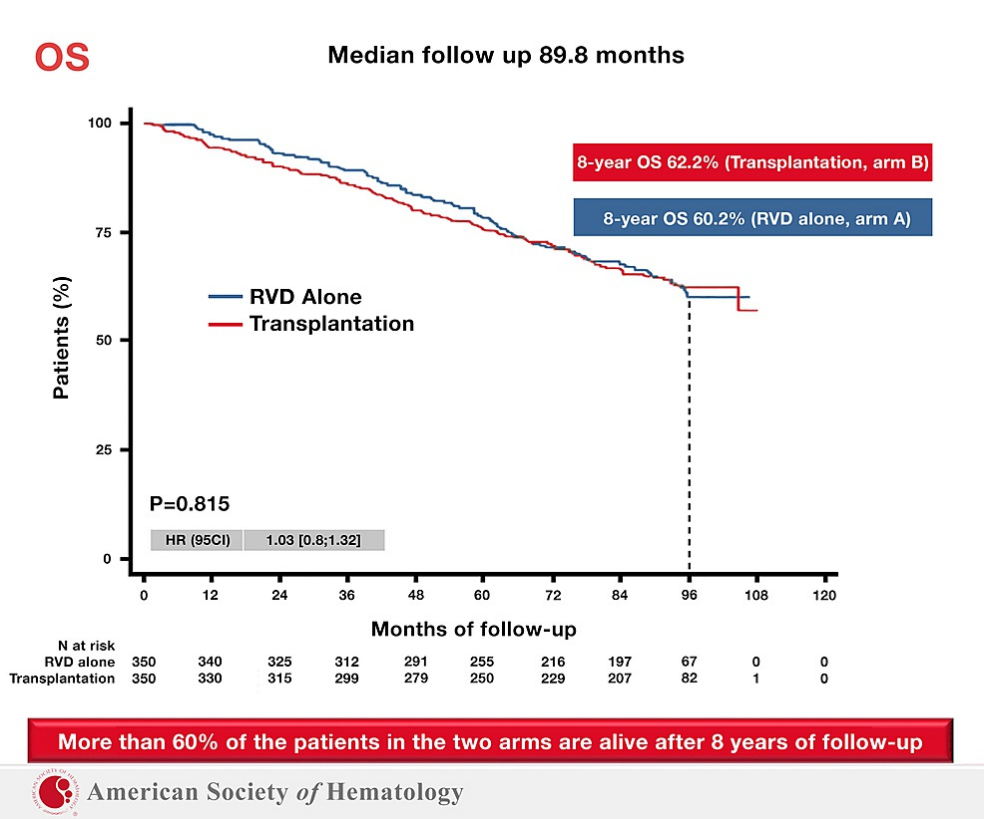
IFM 2009: No Overall Survival Benefit with Stem Cell Transplant After 5.5 Years.

Our analysis brings together information that had not been previously correlated. Although it has previously been shown that the use of alkylating agents in myeloma patients significantly increases the risk of SPM, that difference had curiously not been shown to have a negative impact on survival. Our analysis shows that this negative survival impact does exist but requires six or more years of follow-up in order to become evident. This is likely because it takes time for an SPM to occur, and additional time for that SPM to result in mortality. Smaller studies or studies with shorter follow up can easily miss this effect. A recent update of the FORTE trial advocated for the continued use of stem cell transplants in multiple myeloma patients. However, the study did not yet have six years of follow up by the time it was presented at the American Society of Hematology meeting in 2020 [19]. The IFM 2009 trial also recently reported long-term data in patients treated with lenalidomide, bortezomib, and dexamethasone, with or without stem cell transplant. The overall survival at 8 years was comparable between the transplant and non-transplant cohorts, although progression-free survival (PFS) favored the group that had received SCT (see Figure 2) [20].

IFM 2009 subgroup analysis also showed that progression-free survival was correlated to a patient’s ability to achieve minimal residual disease (MRD) status [20]. However, a significant shortcoming of IFM 2009 is that patients received second- and third-line regimens which are now considered suboptimal, as very few patients were treated with carfilzomib or daratumumab - agents which are considered highly efficacious and now used in early lines of treatment. This potentially resulted in biasing the results in favor of stem cell transplantation, resulting in equivalent overall survival for both arms of the trial.

## Conclusions

Multiple myeloma predisposes patients to the risk of second primary malignancies, and that risk increases in association with multiple factors, including IMiD therapy in patients with previous exposure to alkylating agents. Our analysis is the first to show that the accumulation of second primary malignancies does impact survival over time, with the caveat that long-term follow-up is required in order for that impact to become evident. Recent reports from the FORTE and IFM 2009 trials have asserted that stem cell transplantation still has a role for treatment of patients with multiple myeloma, but the benefits of SCT on overall survival in those studies is nonexistent. Both trials showed improved PFS for patients treated with stem cell transplant, which is believed to be related to an improved percentage of MRD negativity in patients treated with SCT. Based on our results, we suspect that longer-term (6+ years) of follow up in the FORTE trial will be important for determining whether SPMs will negatively impact overall survival in patients treated with SCT, and potentially break the tie in overall survival. As newer agents and non-SCT regimens continue to show improvement in MRD negativity, PFS, and OS, it seems that transplantation for myeloma patients will soon be regarded as unnecessary or even detrimental.
